# Development of Yein-Early, a Unique Fruit-Color and Leaf-Shape Mutant of *Citrus unshiu*, and Its Specific Selection Marker

**DOI:** 10.3390/cimb46090628

**Published:** 2024-09-21

**Authors:** Jung-Gwon Ko, Chang-Ho Eun, In-Jung Kim

**Affiliations:** 1 Faculty of Biotechnology, College of Applied Life Sciences, Jeju National University, Jeju-si 63243, Republic of Korea; dldblb7202@gmail.com; 2 Subtropical Horticulture Research Institute, Jeju National University, Jeju-si 63243, Republic of Korea; 3 Faculty of Biotechnology, College of Applied Life Sciences, Research Institute for Subtropical Agriculture and Biotechnology, SARI, Jeju National University, Jeju-si 63243, Republic of Korea

**Keywords:** citrus, gamma irradiation, SNP, InDel, AS-PCR, molecular marker

## Abstract

*Citrus unshiu* Marc. cv. Miyagawa-wase is one of the most widely cultivated citrus varieties on Jeju Island in Republic of Korea. Mutation breeding is a useful tool for inducing genetic diversity by causing genomic mutations in a short period of time. We previously conducted mutation breeding using gamma irradiation to develop new varieties of *C. unshiu*. Here, we describe one of these varieties, Yein-early, which has a redder peel, greater hardness, and higher sugar content compared with the wild type (WT). Yein-early leaves also showed a unique phenotype compared with the WT, characterized by longer longitudinal length, shorter transverse length, stronger curling, and longer petiole length. Genome resequencing of Yein-early and the WT uncovered significant single-nucleotide polymorphisms (SNPs) and insertions/deletions (InDels). These variations were crucial in identifying molecular markers unique to Yein-early. In addition, we developed an allele-specific PCR marker specifically targeting a homozygous SNP in Yein-early that distinguishes it from the WT and other citrus varieties. This study contributes to the understanding of pigment synthesis in fruits and provides a valuable tool for selection of the novel Yein-early variety in citrus breeding programs.

## 1. Introduction

The “Miyagawa-wase” mandarin (*Citrus unshiu* Marc. cv. Miyagawa-wase) holds a pivotal position in global agriculture. On Jeju Island in Republic of Korea, this variety represents over 80% of citrus production, underscoring its economic and agricultural significance. The breeding of citrus varieties faces numerous challenges, including reproductive complexities such as apomixis, which is a form of asexual reproduction that skips sexual recombination; partial male and female sterility that impacts fruit yield; self- and cross-incompatibility that hinders genetic diversity; a prolonged juvenile period that delays fruit production; and high heterozygosity that leads to variability in offspring [1,2,3]. Furthermore, the seedless nature of Miyagawa-wase, while desirable for consumption, presents additional difficulties in breeding, particularly because of polyembryony, which results in multiple embryos within a single seed, often causing stunted growth of zygotic embryos and complicating selection processes [4]. Moreover, the global use of transgenic citrus plants, which can potentially bypass some of these challenges, is limited by stringent regulations and public perception issues [5].

Mutation breeding, and particularly the use of gamma irradiation, has emerged as a viable solution to address these challenges [6]. Gamma irradiation works by employing gamma rays as a physical mutagen. This process involves the gamma rays interacting with cellular molecules or atoms, leading to the generation of free radicals that can cause chromosomal and DNA damage, thereby increasing the likelihood of beneficial genetic mutations [7]. The application of gamma irradiation is nuanced, with varying effects on plant morphology and physiology depending on the dosage; low doses can stimulate growth and mutation, whereas high doses can lead to growth inhibition or even cell death [8]. Gamma radiation has become a particularly favored method in fruit tree breeding, not only because of its efficacy in inducing mutations but also because of its availability and penetration power and the relative ease with which it can be controlled and applied [9,10,11,12,13].

Our focused research using gamma irradiation on *C. unshiu* Marc. cv. Miyagawa-wase has led to the development of several intriguing mutants. One such mutant, Jedae-unshiu, displays a set of unique and desirable characteristics. These include pronounced vertical troughs on the flavedo, which may contribute to improved aesthetic appeal; a smoother albedo that lacks the rough protruding fibers typical of the wild type (WT); strong adhesion between the peel and the flesh, which enhances the fruit’s structural integrity; and a notable increase in the contents of beneficial flavonoids, including hesperetin and narirutin [14]. These traits offer potential commercial benefits and also provide insights into the genetic pathways that can be influenced by gamma irradiation. Another mutant, Ara-unshiu, shows a late fruit-ripening phenotype, which has significant implications for extending the market availability of the fruit [15]. Our approach has been thorough, encompassing a range of analyses including assessment of morphological traits and examination of sugar and acid contents. Additionally, whole-genome resequencing has been a critical part of our research, allowing us to pinpoint unique molecular markers and understand the genomic changes underlying novel traits.

Mutation, as a process in which genes are permanently altered under environmental conditions and passed between generations, plays a crucial role in the evolution and adaptation of species [16]. Gamma rays are the most widely used mutagen globally and serve as a cornerstone in this process. Sources like Cobalt-60 and Caesium-137 are predominantly used in radiobiological work because of their consistent emission of gamma rays [17]. The application of gamma-ray mutagenesis has been pivotal in citrus breeding and the development of a plethora of new crop varieties across various non-citrus species. Examples include the creation of hardier strains of coriander, the development of new tomato varieties with enhanced traits, the breeding of unique Anthurium flowers, and the improvement of yield in mung bean [18,19,20]. Furthermore, gamma-ray mutagenesis has been instrumental in identifying mutants with significant agricultural implications, such as the semi-dwarf wheat mutant jg0030, which maintains robust yield characteristics [21], and the self-compatible apple mutant Morioka #61-G-827, which represents a breakthrough in apple breeding [22].

Our ongoing research using gamma rays for mutant citrus breeding not only contributes to the development of new, commercially viable, agriculturally beneficial citrus varieties but also significantly enhances our understanding of genetic resources. This underscores the vast potential of mutation breeding in agriculture, offering pathways to overcome breeding challenges, enhance crop traits, and ultimately contribute to sustainable agricultural practices and food security. In the present study, we selected a red-peel and unique leaf-shape mutant, Yein-early, generated from Miyagawa-wase (*C. unshiu*) and investigated its morphological traits and sugar/acid contents. In addition, we conducted whole-genome resequencing to identify a specific molecular marker for the Yein-early mutant.

## 2. Materials and Methods

### 2.1. Plant Materials

We analyzed the Yein-early mutant, generated via gamma-ray exposure, using *C. unshiu* Marc. cv. Miyagawa-wase as the WT control. Both plant materials were cultivated at the Research Institute for Subtropical Agriculture and Biotechnology of Jeju National University, located in Seogwipo, Republic of Korea. The WT control used has been cultivated in the same research institute for a long time. For genome resequencing and transcriptome analysis, we collected young leaves and immature green fruits in July. These samples were quickly chilled in liquid nitrogen to maintain their genetic integrity for future examinations. Other citrus varieties used in this study were Ara-unshiu, Jedae-unshiu, Gownje-early, Chung-Gyeon (*Citrus kiyomi*), Gam-Pyong (*Citrus hybrid* cv. Kanpei), Hanla-Bong (*Citrus reticulata* Shiranui), and Kara-Hyang (*Citrus hybrid* Natsumi).

### 2.2. Analysis of Fruit and Leaf Traits

The analysis of fruit characteristics followed the method of Eun and Kim [14]. Fruit weights were gauged using an electronic scale (CAS Co., Ltd., YangJu, Republic of Korea). The vertical and transverse diameters and peel thickness of the fruit were measured with a digital caliper (MITUTOYO Corporation, Kawasaki, Japan). A fruit hardness meter (LUTRON FR-5105, Antala Staška, Czech) was used to determine fruit firmness. To measure sugar content and acidity, a 4–5 mL sample of fruit juice was analyzed in accordance with the NH-2000 manual (HORIBA, Kyoto, Japan). Peel-color variation was assessed using a CR-400 chromometer (MINOLTA, Tokyo, Japan). To ensure a thorough evaluation, each 65 and 50 fruits were harvested from at least five Yein-early trees and five WT trees each year over 2 years, ensuring a varied sample collection. Leaf morphology analysis was performed by referring to the citrus characteristic guidelines of the Ministry of Agriculture, Food and Rural Affairs and the Republic of Korea Seed Management Institute. Fifteen leaves from each of the three WT individuals and three Yein-early individuals were used to analyze leaf morphology, and the analysis was based on nine leaf characteristics, including longitudinal and transverse lengths, curling, surface undulation, edge waviness, edge indentations, shape of the apex, and grooves at the apex. All statistical analyses were carried out using IBM SPSS software (SPSS for Windows, version 20, SPSS Inc., Armonk, NY, USA). Significant differences among the samples were calculated using an analysis of variance followed by Duncan’s multiple range test at the 5% level (*p* < 0.05).

### 2.3. Whole-Genome Resequencing, Detection of Genetic Variants, and Gene Ontology Analysis

Leaves were harvested from three Yein-early and three WT individuals, and genomic DNA was extracted using the cetyltrimethylammonium bromide method, as described by Healey et al. [23]. These DNAs passed the quality control (QC) process and then sequenced by Macrogen in Daejeon, Republic of Korea, using the Illumina HiSeq X Ten platform. The subsequent steps of sequence pre-processing, alignment to the reference genome, detection, and classification of single-nucleotide polymorphism (SNPs) and insertions/deletions (InDels), and gene ontology (GO) analysis were carried out following the methodologies outlined by Eun and Kim [24]. The reference genome used for this analysis was the *C. unshiu* Marc. Miyagawa-wase assembly (CUMW_v1.0) is available from the National Center for Biotechnology Information (NCBI) and GenBank (accession: GCA_002897195.1). The sequence data generated using this research can be accessed in the NCBI Sequence Read Archive data libraries. The accession numbers for the resequencing datasets are PRJNA745525 (Miyagawa-1) for the WT and PRJNA1046672 for Yein-early.

### 2.4. Allele-Specific PCR Marker

To develop a specific selection marker for the Yein-early mutant, allele-specific PCR (AS-PCR) primers were created, drawing from the guidelines provided by Bui and Lit [24]. Ten homozygous SNPs, identified from the SNP variants between Yein-early and the WT control, were selected for this purpose. For the allele-specific forward primers targeting the Yein-early SNPs, an additional nucleotide mismatch was introduced at the second base position upstream of the 3′ terminus. The reverse primer was designed to have the same sequence for both Yein-early and WT control. For the PCR amplification control, identical forward and reverse primers were used to amplify the PCR product in both Yein-early and the WT. The PCR reactions were conducted in a total volume of 20 µL, using TOPsimple DryMIX-nTaq (Enzynomics, Seoul, Republic of Korea), 5 µM each primer, and 50 ng DNA extract. The cycling conditions were an initial denaturation at 95 °C for 3 min; 30 cycles of 95 °C for 30 s, 57 °C for 30 s, and 72 °C for 15 s; followed by a final extension at 72 °C for 5 min. The PCR products were analyzed by electrophoresis in 2.0% (*w*/*v*) agarose gel. In addition to the DNA samples from Yein-early and the WT control, DNA samples from other citrus varieties were also examined. These varieties included Chung-Gyeon, Gam-Pyong, Hanla-Bong, and Kara-Hyang.

## 3. Results

### 3.1. Selection of Mutant Lines by Gamma Irradiation

Gamma irradiation was used to develop mutant citrus lines, leading to the creation of the Yein-early mutant. This process involved exposing *C. unshiu* scions to gamma rays and then grafting them onto Miyagawa-wase branches, resulting in various mutant lines. Among these, Yein-early, distinguished by its red fruit color, was selected for further study. Subsequent grafting and consistent monitoring confirmed the stable expression of this trait [25,26].

### 3.2. Morphological Differences between Yein-Early and WT Fruits and Leaves

**Figure 1:**
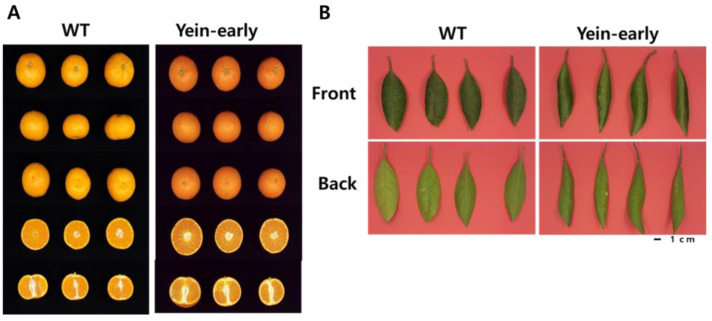
Morphological comparison of fruits (**A**) and leaves (**B**) between Yein-early and wild type (WT).

We conducted a comparative morphological analysis of the external physical attributes of Yein-early and WT fruits. Yein-early fruits displayed a distinct red peel coloration, markedly contrasting with the orange color of the WT fruits (Figure 1A). Over a 2-year observational period, the Yein-early fruits had significantly greater hardness than the WT fruits (Yein-early: 1299.03 ± 168.76; WT: 876.48 ± 112.94), whereas the size, weight, and peel thickness were comparable between the two varieties (Table 1). Hunter color values showed a higher red index for Yein-early fruits (Yein-early: 33.33 ± 2.23; WT: 25.79 ± 1.84), without significant difference in the white and yellow indices. In addition, Yein-early fruits had a significantly higher sugar content (Yein-early: 10.39 ± 0/97; WT: 9.40 ± 0.30), with acidity levels similar to those of WT fruits (Table 2). In terms of leaf phenotypes, Yein-early leaves were longer (Yein-early: 108.77 ± 7.14; WT: 95.44 ± 7.58) and narrower (Yein-early: 35.02 ± 4.69; WT: 42.50 ± 4.52) than WT leaves, and their cross-sections exhibited a marked concavity with increased curling. All other phenotypic characteristics were consistent with those of the WT (Table 3; Figure 1B).

### 3.3. Mapping of Sequencing Reads to the Reference Genome

To delve deeper into the source of the red fruit color of Yein-early, we performed whole-genome sequencing to identify DNA sequence polymorphisms between Yein-early and the WT. The WT sequence data were sourced from our previously published work (NCBI: PRJNA745525) [27]. For a reference, we used the *C. unshiu* Marc. Miyagawa-wase (CUMW_v1.0) genome, which is 359.7 Mb in length [28]. Post-sequencing pre-processing yielded 55,897 Mbp and 56,418 Mbp of clean reads for Yein-early and the WT, respectively. The mapping rates on the reference genome were 84.80% for Yein-early and 86.85% for the WT (Table 4). In comparison with the reference genome, we detected 1,185,693 SNPs in the WT and 1,583,458 SNPs in Yein-early (Table S1). Among these, the WT showed 2893 homozygous and 767,018 heterozygous SNPs, and Yein-early showed 304,513 homozygous and 772,143 heterozygous SNPs. These SNPs were further classified by genome annotation (Table S2), revealing 364,278 SNPs in genic regions (161,724 in exons and 212,199 in introns) and 745,808 SNPs in intergenic regions for the WT. In Yein-early, 456,272 SNPs were detected in genic regions (203,235 in exons and 265,470 in introns), and 994,979 SNPs were detected in intergenic regions.

InDel detection in the WT and Yein-early genomes also yielded significant results in comparison with the reference genome (Table S3). In the WT, the total number of InDels was 282,179, of which 12,427 were homozygous (4259 insertions and 8168 deletions) and 126,723 were heterozygous (61,006 insertions and 65,717 deletions). Yein-early showed a total of 385,594 InDels, of which 40,890 were homozygous (17,426 insertions and 23,464 deletions) and 197,671 were heterozygous (97,973 insertions and 99,698 deletions). In the WT, 256,036 of the InDels could be annotated based on the reference genome, showing that 60,222 were in genic regions (4928 in CDS, 14,809 in exons, and 47,003 in introns) and 195,814 were in intergenic regions. In Yein-early, 352,308 InDels could be annotated, showing that 78,968 were in genic regions (6528 in CDS, 19,444 in exons, and 61,643 in introns) and 273,340 were in intergenic regions (Table S4).

### 3.4. Genetic Variation between the Wild Type and Yein-Early

To identify the mutation responsible for the red fruit color of Yein-early, we conducted a comparative genomic analysis between the WT and Yein-early. This revealed a substantial number of genetic differences: 650,257 SNPs (72,155 homozygous and 578,102 heterozygous) and 105,817 InDels (4683 homozygous and 101,134 heterozygous; Table 5). Further scrutiny of the SNPs and InDels identified a total of 650,257 SNPs and 105,817 InDels in 26,339 and 14,889 genes, respectively, for which GO annotation was available in the Coding DNA Sequences database (referenced in Table 6). By comparing the genetic variation between the two samples, it was possible to filter out many non-specific SNPs and InDels. This process highlighted that specific SNPs or InDels unique to Yein-early could be identified by comparison with the WT control. The findings indicated that the genome of Yein-early is more prominently mutated with SNP polymorphisms than with InDels. This contrasts with the genome resequencing of the Jedae-unshiu mutant, in which InDels were more prevalent than SNPs, as reported by Eun and Kim in 2022 [14]. We used the Plant Transcription Factor (TF) Database http://planttfdb.gao-lab.org/ (accessed on 13 July 2023) and Google Scholar to search for TFs among the homozygous SNPs and InDels with sequencing depths exceeding 20 and 10, respectively. This search identified three homozygous SNPs and two homozygous InDels that were located within TFs. The three TFs with homozygous SNPs were FAR1-RELATED SEQUENCE 5 (FAS5), L10-interacting MYB domain-containing protein (LIMYB), and FAR1-RELATED SEQUENCE-like (FAS-L). The two TFs with homozygous InDels were wall-associated receptor kinase (WARK)-like 10 isoform X3 and wall-associated receptor kinase-like 22 (Table S5). We next investigated the amino acid changes resulting from the five TF mutations. We found that the SNP in FAS5 resulted in a change from valine in the WT to isoleucine in Yein-early at amino acid position 393 (Figure S1). The amino acid changes associated with the remaining two SNPs and the two InDels in TFs could not be confirmed, because there was no full-length sequence available in the NCBI database.

### 3.5. Functional Annotation of Non-Synonymous Mutations in Yein-Early

**Figure 2:**
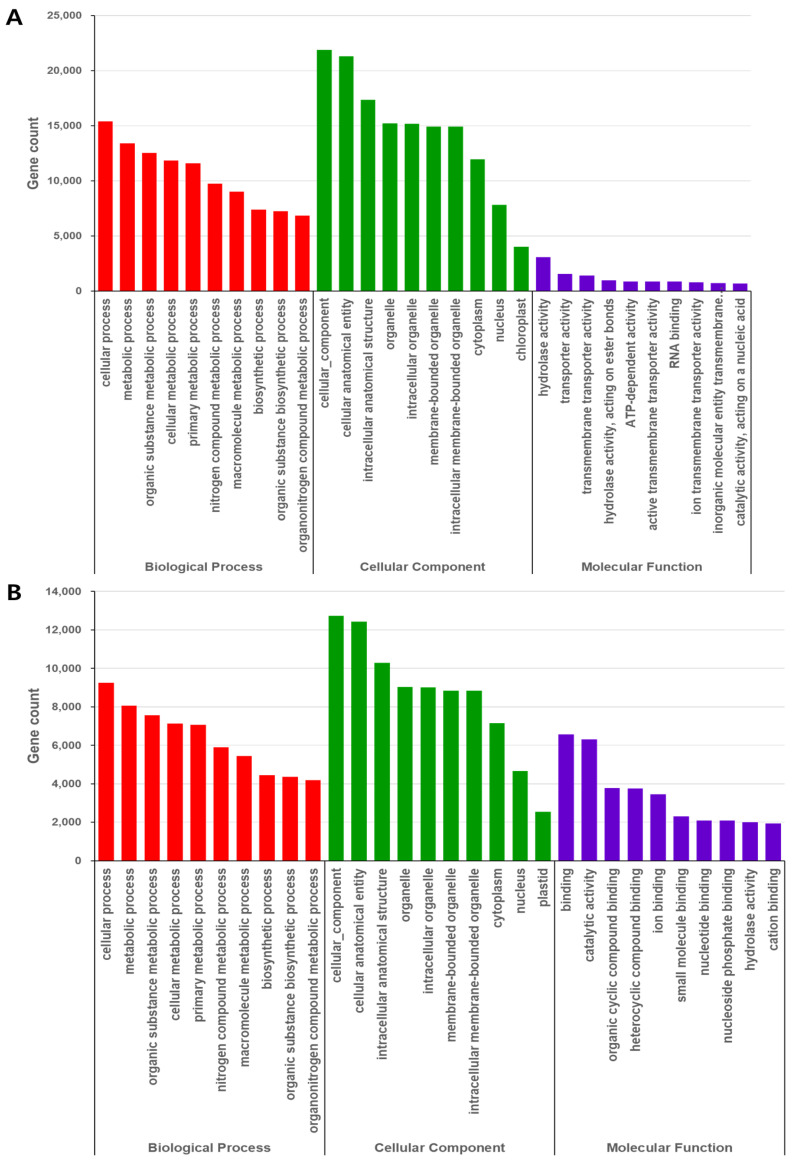
Gene Ontology functional enrichment of variant genes containing SNPs and InDels (WT vs. Yein-early). (**A**) Top 20 Biological Process, Cellular Component, and Molecular Function subcategories attributed to the genes containing SNPs. (**B**) Top 10 Biological Process, Cellular Component, and Molecular Function subcategories attributed to the genes containing InDels.

We meticulously annotated the non-synonymous SNPs and InDels in the coding sequence of Yein-early using the GO database, with a focus on gene function alterations. We systematically classified the mutated genes into three main functional categories: Biological Process (BP), Cellular Component (CC), and Molecular Function (MF). Among the genes containing SNPs, 429,369 had BP functions, which were further classified into 501 subcategories, including cellular process (15,397 genes), metabolic process (13,394 genes), organic substance metabolic process (12,518 genes), cellular metabolic process (11,826 genes), and primary metabolic process (11,589 genes). A total of 157,544 genes containing SNPs had CC functions, which included 29 subcategories such as cellular component (21,892 genes), cellular anatomical entity (21,294 genes), intracellular anatomical structure (17,363 genes), organelle (15,205 genes), and intracellular organelle (15,192 genes). A total of 20,836 genes containing SNPs had MF functions. These could be further grouped into 43 subcategories, including hydrolase activity (3,065 genes), transporter activity (1562 genes), transmembrane transporter activity (1,419 genes), hydrolase activity on ester bonds (988 genes), and ATP-dependent activity (868 genes). Among the genes containing InDels, 319,632 had BP functions, including cellular process (9238 genes), metabolic process (8068 genes), organic substance metabolic process (7551 genes), cellular metabolic process (7131 genes), and primary metabolic process (7056 genes). A total of 103,751 genes containing InDels had CC functions, which could be further divided into 100 subcategories, including cellular component (12,727 genes), cellular anatomical entity (12,427 genes), intracellular anatomical structure (10,295 genes), organelle (9026 genes), and intracellular organelle (9017 genes). Finally, 66,768 genes containing InDels had MF functions, which included 169 subcategories such as binding (6558 genes), catalytic activity (6315 genes), organic cyclic compound binding (3779 genes), heterocyclic compound binding (3765 genes), and ion binding (3453 genes; Figure 2).

### 3.6. Identification of a Specific Selection Marker for Yein-Early

**Figure 3:**
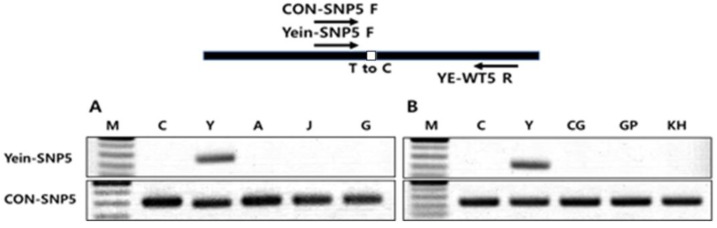
Yein-early selection by allele-specific PCR. (**A**) C: WT control; Y: Yein-early; A: Ara-unshiu; J: Jedae-unshiu; G: Gownjae-early. (**B**) C: WT control; Y: Yein-early; J: Jedae-unshiu; CG: Chung-Gyeon; GP: Gam-Pyong; KH: Kara-Hyang. A, J, G: our other citrus variants; CG, GP, KH: commercially available citrus varieties.

AS-PCR is a method for SNP detection in which the extension of a PCR primer occurs exclusively when the 3′ end of the primer is in complete alignment with the template sequence [29,30]. To explore mutation-specific selection markers for Yein-early using AS-PCR, we selected 10 homozygous SNPs between Yein-early and the WT and designed a common primer and an allele-specific primer for each, the latter featuring an intentional mismatch near the SNP locus (Table S5). Among these 10 primer pairs, one pair, Yein-SNP5, demonstrated efficacy in distinguishing Yein-early from the WT and also from other mutant varieties and citrus varieties (Figure 3).

## 4. Discussion

The development of the Yein-early mutant of *C. unshiu* marks a pivotal breakthrough in citrus breeding that is particularly advantageous for regions like Jeju Island. This mutant, characterized by its unique red peel, not only has enhanced aesthetic appeal but may also harbor changes in phytochemical composition that improve fruit quality and provide health benefits. This mirrors the characteristics of the Jedae-unshiu mutant, another variety developed through gamma irradiation, which is known for its increased flavonoid content and unique fruit shape [14]. Moreover, the increased hardness and higher sugar content of Yein-early align with consumer preferences, suggesting its potential as a premium citrus variety. This success is reminiscent of the development in Japan of various *C. unshiu* cultivars using conventional methods [31]. The significant challenges associated with traditional breeding of citrus varieties, such as apomixis and long seedling periods, underscore the effectiveness of gamma irradiation as an alternative technique. This is further supported by advancements in genomics and genome editing, which have the potential to accelerate breeding processes and introduce beneficial traits in a manner similar to that of natural plant mutations [32]. Recently, research has been conducted to develop mutants with excellent functionality in citrus fruits. Wang et al. identified *Aiyuan 38*, a yellowish-bud mutant that produces pulp containing six unique volatile organic compounds compared with the WT [33]. *Langfeng*, a novel bud mutant of navel orange (*Citrus sinensis*), has tolerance to chlorosis in acidic and magnesium-deficient soils [34]. By exposing seeds of *Citrus Macrophylla* to gamma rays, Pérez-Jiménez et al. obtained a salt-tolerant mutant, MM5B, that suffers less leaf damage and contains higher proline levels than the WT under conditions of salt stress [35]. Kozan Yerli, a seedless mutant of the seedy “Kozan” common sweet orange (*C. sinensis* (L.) Osb.), was obtained by exposing autumn shoots to gamma irradiation from ^60^Co [36].

In the Yein-early mutant, the search for TFs by filtering homozygous SNPs and InDels with a sequencing depth exceeding 20 and 10, respectively, led to the identification of three SNPs and two InDels (Table S5). Among the three TFs containing SNPs, *FAR1-RELATED SEQUENCE* is known as a transposase-derived TF with crucial functions in plant growth and development, light signaling transduction, phytohormone response, and stress resistance [37,38,39]. LIMYB links leucine-rich repeat receptor-like kinase immune-receptor activation to global translation suppression as an antiviral immunity strategy in plants [40]. LIMYB was also identified as one of the seven hub genes with high connectivity in gene co-expression modules associated with flowering development time in *Elymus sibiricus* [41]. FAS-L has crucial roles in plant growth and development in Arabidopsis [38]. Among the two TFs containing InDels, WARK-like 10 isoform X3 is known to function as a negative regulator of leaf senescence and has been associated with defense and hormone responses [42]. Wall-associated kinase-like 22 was reported to be associated with activation of plant defense mechanisms in response to *Botrytis cinerea* infection in Rosa chinensis, suggesting its potential to mediate plant resistance to pathogens [43].

Developments in mutation breeding, particularly using gamma irradiation, have been instrumental in developing new plant varieties. Gamma irradiation is effective in inducing significant trait changes, as evidenced in the Jedae-unshiu mutant, and can be used across various citrus species, including lemons, mandarins, and limes [15,44]. Research on the “Yaghouti” grape offers valuable insights into the dose-dependent effects of gamma irradiation, which are crucial for understanding its effects on mutants like Yein-early [45]. The development of Yein-early highlights the potential of mutation breeding to help meet the global demand for high-quality, easy-to-peel citrus cultivars [46] and also aligns with previous contributions of natural mutations in the citrus industry [47]. The use of an allele-specific PCR marker for Yein-early can be combined for rapid development and introduction of new citrus varieties with traditional breeding methods like inter- and intra-specific crosses and clonal selection, which have led to the development of several promising varieties [48]. The development of the Yein-early mutant underscores the efficacy of gamma irradiation in inducing beneficial mutations in *C. unshiu*, paving the way for future research in citrus mutagenesis and breeding to develop new fruit varieties that cater to specific consumer needs and market trends. Future research should focus on understanding the long-term stability of Yein-early traits, their effects on fruit quality, and their underlying molecular mechanisms. To investigate the molecular mechanisms responsible for the redder peel and higher sugar content of the Yein-early compared to the WT, we plan to perform transcriptome and metabolite analysis along fruit development from immature fruit in July to full maturity in December.

## Figures and Tables

**Table 1:** Comparative analysis of wild-type (WT) and Yein-early fruits.

	Year	Vertical (mm)	Horizontal (mm)	Weight (g)	Peel Thickness (mm)	Hardness (G)
WT	2021	46.27 ± 3.89	58.45 ± 4.03	87.60 ± 13.73	2.12 ± 0.22	937.57 ± 58.37
	2022	47.83 ± 3.68	62.32 ± 6.64	98.94 ± 29.29	2.65 ± 0.43	862.38 ± 119.28
Average		47.54 ± 3.64	61.60 ± 6.32	96.81 ± 27.06	2.55 ± 0.45	876.48 ± 112.94
Yein-early	2021	51.27 ± 3.20	59.35 ± 3.22	91.76 ± 14.19	2.84 ± 0.36	1263.24 ± 200.50
	2022	54.60 ± 2.48	62.61 ± 3.46	113.51 ± 15.56	3.81 ± 0.73	1318.31 ± 154.41
Average		53.43 ± 3.13	61.47 ± 3.66	105.90 ± 18.16	3.47 ± 0.78	1299.03 ± 168.76 *

* Indicates a value with a significant difference compared to the control (*p* < 0.05). G: force of gravity.

**Table 2:** Comparative analysis of sugar, acidity, and Hunter color value between wild-type (WT) and Yein-early fruits.

	Year	Sugar (Brix)	Acidity (wt%)	Hunter Color Value		
				L	a	b
WT	2021	9.38 ± 0.27	0.72 ± 0.08	59.36 ± 1.50	25.22 ± 0.72	35.47 ± 0.59
	2022	9.41 ± 0.32	0.46 ± 0.03	59.47 ± 1.62	25.92 ± 2.01	35.28 ± 1.05
Average		9.40 ± 0.30	0.51 ± 0.11	59.45 ± 1.55	25.79 ± 1.84	35.32 ± 0.96
Yein-early	2021	10.28 ± 0.59	0.91 ± 0.09	56.25 ± 1.00	34.31 ± 1.83	32.70 ± 0.77
	2022	10.45 ± 1.15	0.81 ± 0.11	55.63 ± 2.37	32.80 ± 2.31	32.36 ± 1.72
Average		10.39 ± 0.97 *	0.85 ± 0.11	55.85 ± 1.99	33.33 ± 2.23 *	32.48 ± 1.44

* Indicates a value with a significant difference compared to the control (*p* < 0.05).

**Table 3:** Comparative analysis of wild-type (WT) and Yein-early leaves.

	WT	Yein-Early
Transverse Length	95.44 ± 7.58	108.77 ± 7.14 *
Longitudinal Length	42.50 ± 4.52	35.02 ± 4.69 *
Longitudinal/Transverse	2.26 ± 0.16	3.15 ± 0.44 *
Curling	weak	strong
Surface Undulation	weak	weak
Edge Waviness	weak	weak
Edge Indentations	blunt saw, blade shape	blunt saw, blade shape
Shape of the Apex	sharp	sharp
Groove at the Tip	no	no

* Indicates a value with a significant difference compared to the control (*p* < 0.05).

**Table 4:** Statistics for sequencing data in WT and Yein-early.

Sample	Clean Reads ^1^	Mapped Reads ^2^	Mapped Region ^3^ (%)
WT	56,418,140	55,183,019 (97.81%)	312,343,061 (86.85%)
Yein-early	55,897,538	54,686,868 (97.83%)	304,277,009 (84.60%)

^1^ No. of total trimmed reads: Total number of clean reads used for read alignment after passing pre-processing. ^2^ The number of aligned clean reads mapped to the reference sequence. ^3^ Base pairs of the region covered by read alignment compared to the reference sequence.

**Table 5:** Statistics of polymorphic SNPs and InDels between the WT and Yein-early.

Sample		No. of Total	No. of Polymorphic	
		Polymorphic ^1^	Homo ^2^	Hetero ^3^
WT vs. Yein-early	SNP	650,257	72,155	578,102
	InDel	105,817	4683	101,134

^1^ SNP and InDel loci showing differences in nucleotide sequences between comparative samples. ^2^ When the nucleotide sequences of both samples are homozygous. ^3^ When the nucleotide sequence is homozygous in one sample and heterozygous in the other sample.

**Table 6:** Statistics of Gene Ontology (GO) annotations for polymorphic SNPs and InDels between the WT and Yein-early.

Sample		Polymorphic	Genes ^1^	GO Genes ^2^
WT vs. Yein-early	SNP	650,257	26,339	23,508
	InDel	105,817	14,889	13,685

^1^ Number of genes corresponding to SNP or InDel loci. ^2^ Number of genes annotated in the GO database among the genes corresponding to SNP or InDel loci (e-value ≤ 1 × 10^−10^ best hits).

## Data Availability

Original sequence data can be found in the NCBI Sequence Read Archive with the following accession numbers: PRJNA745525 for WT (Miyagawa-1) and PRJNA1046672 for Yein-early. Other data are available upon request.

## Supplementary Materials

The following supporting information can be downloaded at: https://www.mdpi.com/article/10.3390/cimb46090628/s1, Figure S1: Comparison of the amino acid sequences of FAR1-RELATED SEQUENCE 5 between WT and Yein-early; Table S1: Statistics of SNP detection in samples; Table S2: Statistics of SNP classification by genome annotation; Table S3: Statistics of InDel detection in samples; Table S4: Statistics of InDel classification by genome annotation; Table S5: Transcription factors with homozygous SNPs and InDels; Table S6: SNP primer sequences used in this study.
