# Supplementary material for: Development of Yein-Early, a Unique Fruit-Color and Leaf-Shape Mutant of Citrus unshiu, and Its Specific Selection Marker
Source: Curr Issues Mol Biol. 2024 Sep 21;46(9):10606–17. doi: 10.3390/cimb46090628 (PMC11430535; doi:10.3390/cimb46090628)
Supplement: Supplementary file 1 [file cimb-46-00628-s001.zip › cimb-3195282-supplementary.pdf]

Supplementary

Table S1. Statistics of SNP detection in samples

Sample	Total SNPs	Homozygous[1]	Heterozygous[2]	Other[3]
Control	1,185,693	2,893	767,018	415,782
Yein-early	1,583,458	304,513	772,143	506,80

[1] The same SNP type showed in 90% of sample reads mapped to the reference genome

[2] The same SNP type showed in 40%~60% of sample reads mapped to the reference genome

[3] Not applicable as homozygous/heterozygous

Table S2. Statistics of SNP classification by genome annotation

Sample	Total SNPs	Annotated SNPs[1]	IGR[2] & Gene Structure	Total	Homozygous	Heterozygous	Other[3]
WT	1,185,693	1,080,086	intergenic region	745,808	1,720	454,333	259,755
			genic region	364,278	486	454,333	107,075
			exon	161,724	308	112,512	48,904
			intron	212,199	192	150,771	61,236
Yein-early	1,193,106	1,078,912	intergenic region	994,979	195,825	472,230	326,924
			genic region	456,272	78,296	249,768	45,701
			exon	203,235	35,194	109,730	58,311
			intron	265,470	45,261	146,653	73,556

[1] Loci where genes are organized in a reference gene annotation (gff) file and can be classified as genic/intergenic

[2] Intergenic region

[3] Not applicable as homozygous/heterozygous

Table S3. Statistics of InDel detection in samples

Sample	Total		Homozygous[1]		Heterozygous[2]		Other[3]	
	Total	Insertions/Deletions	Total	Insertions/Deletions	Total	Insertions/Deletions	Total	Insertions/Deletions
WT	282,178	136,488 / 145,690	12,427	4,259/8,168	126,723	61,006/65,717	143,028	71,223/71,805
Yein-early	385,594	185,496 / 200,098	40,890	17,426/23,464	147,033	70,097/76,936	197,671	97,973/99,698

[1] The same InDel type showed in 90% of sample reads mapped to the reference genome

[2] The same InDel type showed in 40%~60% of sample reads mapped to the reference genome

[3] No applicable as the homozygous/heterozygous

Table S4. Statistics of InDel classification by genome annotation

Sample	Total InDels	Annotated InDels[1]	IGR[2] & Gene Structure	Total	Homozygous	Heterozygous	Other[3]
WT	282,178	256,036	intergenic region	195,814	8,459	87,186	100,169
			genic region	60,222	1,429	30,148	28,645
			CDS	4,928	359	2,048	2,521
			exon	14,809	570	7,222	7,017
			intron	47,003	922	23,775	22,306
Yein-early	385,594	352,308	intergenic region	273,340	28,455	103,746	141,139
			genic region	78,968	7209	33,516	38,243
			CDS	6,528	717	2,309	3,502
			exon	19,444	1943	8,030	9,471
			intron	61,643	5494	26,416	29,733

[1] Loci where genes are organized in a reference gene annotation (gff) file and can be classified as genic/intergenic

[2] Intergenic region

[3] Not applicable as the homozygous/heterozygous

※ If the reference gene position overlaps or due to the anti-sense form of the gene, the number of exons and introns may be counted as duplicates.

Table S5. Transcription factors with homozygous SNPs and InDels

Type	ID	Position	WT	Yein-early	Description
SNP	scaffold00430	109464	A	G	protein FAR1-RELATED SEQUENCE 5
SNP	scaffold00194	37085	T	C	L10-interacting MYB domain-containing protein
SNP	scaffold00899	8530	A	G	protein FAR1-RELATED SEQUENCE 5-like
InDel	scaffold00072	742016	*	+1G	wall-associated receptor kinase-like 10 isoform X3
InDel	scaffold00072	745918	*	-+1T	wall-associated receptor kinase-like 22

Table S6. SNP primer sequences used in this study

Primer name	Sequence(5`-3`)	Length	Tm	GC contents
Yein-SNP1 F	5- CGGAGGAAACGATGTAACTACTG -3	23bp	60.1℃	47.8%
Con-SNP1 R	5- TCCATCCTGCAAAGTCAACA -3	20bp	60.2℃	45.0%
Yein-SNP2 F	5-CCGCGTACTGTTGACTGATAAT-3	22bp	59.2℃	45.5%
Con-SNP2 R	5-TCATAATTGAACTAGGGGCAAAA-3	23bp	59.9℃	34.8%
Yein-SNP3 F	5- AGAGCTAAGAGGAAAACCAGAAATT-3	25bp	59.4℃	36.0%
Con-SNP3 R	5- ATCACCAACGCAACTTCCTC -3	20bp	60.1℃	50.0%
Yein-SNP4 F	5- GTTTGGAGATGTCTTGTAGAAGGAC -3	25bp	59.6℃	44.0%
Con-SNP4 R	5- TCCCCGATAGTGTCTTGAATG -3	21bp	59.9℃	47.6%
Con-SNP5 F	5- GCTGAAAGGCAACAAATGAACT-3	22bp	60.7℃	40.90%
Yein-SNP5 F	5- CTGAAAGGCAACAAATGAACC-3	21bp	59.6℃	42.90%
Con-SNP5 R	5- TCGCCCATTAAAGTCTCCAC -3	20bp	60.1℃	50.00%
Yein-SNP6 F	5- CATAAAAATGGTCTGTTGCACAG-3	23bp	59.6℃	39.1%
Con-SNP6 R	5- TAGGACCACTTTTGGCAAGC -3	20bp	60.2℃	50.0%
Yein-SNP7 F	5- AGGCTTCAAGCTTGATTGTCA-3	21bp	60.0℃	42.9%
Con-SNP7 R	5- TTTCGGTCGCTGAATGTGTA-3	20bp	60.3℃	45.0%
Yein-SNP8 F	5- CGAGTACACATTCAGCGATCA -3	21bp	59.9℃	47.6%
Con-SNP8 R	5- CATGGCCCGTGGTCTATAAT -3	20bp	59.7℃	50.0%
Yein-SNP9 F	5- GGAGAAAGTTGAGCTTGATCTGATT -3	25bp	61.4℃	40.0%
Con-SNP9 R	5- TGCTGTTTAGCTTGGTAGGC -3	20bp	58.2℃	50.0%
Yein-SNP10 F	5- CCCAATAGATTTGTCCATGCAG-3	22bp	61.6℃	45.5%
Con-SNP10 R	5- CCAACATAATTCGGGAGGTG -3	20bp	60.2℃	50.0%

```
                                  20                    40                    60
C.unshiu    MPFAPFVGVN HHGHSILFGC GLISNEDIET FTWLFRTWLS CMSNSAPIGI ITDQDKAMKA AIENVFPNTR 70
Yein-early  MPFAPFVGVN HHGHSILFGC GLISNEDIET FTWLFRTWLS CMSNSAPIGI ITDQDKAMKA AIENVFPNTR 70
                       80                   100                   120                   140
C.unshiu    HRWCLWHILK KIPEKLGGYK EYRDISNVLH CAVYDSQSSA KFEETWHHMI VEFELGGNEW LRGLYNERHR 140
Yein-early  HRWCLWHILK KIPEKLGGYK EYRDISNVLH CAVYDSQSSA KFEETWHHMI VEFELGGNEW LRGLYNERHR 140
                                 160                   180                   200
C.unshiu    WVPCYLKNSF WAGMSTTQRS ESMNAFFDGY VNSKTTLKQF VEQYSNALKN KAQKEVEEDA RCSSHQLPCV 210
Yein-early  WVPCYLKNSF WAGMSTTQRS ESMNAFFDGY VNSKTTLKQF VEQYSNALKN KAQKEVEEDA RCSSHQLPCV 210
                      220                   240                   260                   280
C.unshiu    TSYAMEKQIR DVYTISKFQE FQQELVGKMY CEFVNSMGCE YIVREDVKVD TWKKRTFFEV YCDKENGEIH 280
Yein-early  TSYAMEKQIR DVYTISKFQE FQQELVGKMY CEFVNSMGCE YIVREDVKVD TWKKRTFFEV YCDKENGEIH 280
                                 300                   320                   340
C.unshiu    CSCSRFQFRG ILYRHAITIM IRNDMEVLPE KFILRRWRKD VWRCHSRVKT SHELHSCTDE QKRYEKMCVS 350
Yein-early  CSCSRFQFRG ILYRHAITIM IRNDMEVLPE KFILRRWRKD VWRCHSRVKT SHELHSCTDE QKRYEKMCVS 350
                      360                   380                   400                   420
C.unshiu    FAEVANVAAK DVESYNLVLN WIEKVRIDLP ETIQCGGNDV TIVTGQGSCS NSVGIEMHDV ETVRDPVVKR 420
Yein-early  FAEVANVAAK DVESYNLVLN WIEKVRIDLP ETIQCGGNDV TIITGQGSCS NSVGIEMHDV ETVRDPVVKR 420
                                 440                   460                   480
C.unshiu    RKCRPPCQRK KSTKSSKSAR SNAKDGHVSD QVATMIPTFQ NLQQSSVDRN MYPHGVMDYS NGLMNLHHIT 490
Yein-early  RKCRPPCQRK KSTKSSKSAR SNAKDGHVSD QVATMIPTFQ NLQQSSVDRN MYPHGVMDYS NGLMNLHHIT 490
                      500                   520                   540
C.unshiu    GDNVYPTATQ NSSTHPQLQP QVHGHFQQLL FHQNNEYGVN REFGSDFEE* 540
Yein-early  GDNVYPTATQ NSSTHPQLQP QVHGHFQQLL FHQNNEYGVN REFGSDFEE* 540
```

Figure S1. Comparison of the amino acid sequences of FAR1-RELATED SEQUENCE 5 between WT and Yein-early.
